# Plant- vs. Bacterial-Derived Cellulose for Wound Healing: A Review

**DOI:** 10.3390/ijerph17186803

**Published:** 2020-09-18

**Authors:** Ruth Naomi, Ruszymah Bt Hj Idrus, Mh Busra Fauzi

**Affiliations:** 1 Centre for Tissue Engineering and Regenerative Medicine, Faculty of Medicine, Universiti Kebangsaan Malaysia, Cheras, Kuala Lumpur 56000, Malaysia; ruthmanuel2104@gmail.com (R.N.); ruszyidrus@gmail.com (R.B.H.I.); 2 Department of Physiology, Faculty of Medicine, Universiti Kebangsaan Malaysia, Cheras, Kuala Lumpur 56000, Malaysia

**Keywords:** plant cellulose, bacterial cellulose, wound healing, clinical trials, in vivo, in vitro

## Abstract

Cellulose is a naturally existing element in the plant’s cell wall and in several bacteria. The unique characteristics of bacterial cellulose (BC), such as non-toxicity, biodegradability, hydrophilicity, and biocompatibility, together with the modifiable form of nanocellulose, or the integration with nanoparticles, such as nanosilver (AgNP), all for antibacterial effects, contributes to the extensive usage of BC in wound healing applications. Due to this, BC has gained much demand and attention for therapeutical usage over time, especially in the pharmaceutical industry when compared to plant cellulose (PC). This paper reviews the progress of related research based on in vitro, in vivo, and clinical trials, including the overall information concerning BC and PC production and its mechanisms in wound healing. The physicochemical differences between BC and PC have been clearly summarized in a comparison table. Meanwhile, the latest Food and Drug Administration (FDA) approved BC products in the biomedical field are thoroughly discussed with their applications. The paper concludes on the need for further investigations of BC in the future, in an attempt to make BC an essential wound dressing that has the ability to be marketable in the global marketplace.

## 1. Introduction

The rapid emergence of essential bio-based material production and development has tremendously intrigued substantial interest in investigating cellulose as a large natural source for various applications, including for its use in medicines. To date, cellulose is widely known for its unique properties, such as biocompatibility, hydrophilicity, and non-toxicity, as well as for its use in antimicrobial and hemostatic agents. Due to the previously mentioned unique properties, the cost for cellulose in the global marketplace has gradually increased. By the year 2026, it is expected that the global market price for cellulose could reach up to USD 305.08 billion [1]. The highest usage of cellulose in this era is expected to be from the pharmaceutical and engineering industries. The growth of the cellulose market is driven by the production of cellulose-based products from top-down (plant cellulose (PC)-derived products), or bottom-up (bacterial cellulose (BC)-derived products) approach. The Asia Pacific region was the highest in revenue for the cellulose market in the year 2015 [1], valued at approximately USD 7.4 billion [2]. Based on cellulose derivatives, these can be segmented into commodities that include cellulose fibers, cellulose ethers, microcrystalline cellulose, cellulose pulp, and other similar derivatives. Thus, the renewability and the sustainability of bio-based products, such as these cellulosic materials, are profoundly employed to use available resources with higher efficiency.

Cellulose is a polysaccharide that has the most readily available biopolymer in nature for over a thousand years. It is the most essential molecule of a plant that has been identified as the major component of a cell wall. Cellulose is glucose in a linear form, which is also known as anhydroglucose, and this is linked together by β-1,4, in means of the glucose residues [3,4,5]. The linear structure of cellulose is well-maintained in the form of a cellulose chain, due to the presence of a hydrogen bond that holds the oxygen atom and hydroxyl together [3,5]. This structure enables it to be used for various biochemical needs.

In the therapeutic field, bioactivity, biocompatibility, and biomechanics are the three main components that will be considered for any biomaterial. In accordance with this, cellulose is naturally biocompatible to human tissue, as it is made up of a polysaccharide chain being a polymer of the glucose subunits. This can easily be modified, without altering its structural or mechanical properties. Cellulose is able to exhibit biological effects upon its modification, due to its semi-crystalline property in an aqueous state. These characteristics make cellulose easy to bind and to be tableted in any form, due to its cohesiveness property in a moist microenvironment. At the same time, the presence of a cell wall contributes to the biomechanics property of cellulose. Being a natural polymer, cellulose is widely available in plants and in the continuous photosynthesize processes in plants, to enable the production of 1011 ± 1012 tons of pure cellulose. In India, cellulose makes up around 50% of all current wastes. Although cellulose is not degradable in humans, it still can assist in defecation, due to its hydrophilic property. All these mentioned properties satisfy all of the components needed for biomedical needs, and these serve as the main reason for the incorporation of cellulose in tissue engineering [4,6]. In this review, we focused on the physicochemical property of the bacterial and plant derived cellulose, regardless of its further modification; that accelerates the wound healing process. Moreover, in vitro, in vivo, and FDA approved PC and BC products will be briefly explained further to support the main idea of this review. The article concluded on the importance of further potential research on PC and BC in order to incorporate both in therapeutic field effectively.

## 2. Search Strategy

The search strategy for this review was done from five electronic database: EBSCOhost, ScienceDirect, PubMed, Medline and Ovid and Web of Science. The search was done from year 2008 up to May 2020. A full update was performed on 1 June 2020. The search query consists of keywords such as plant cellulose, bacterial cellulose, in vitro, in vivo, structure, properties, wound healing, importance and medical application.

## 3. Cellulose Structure

Cellulose is made up of four main components, namely, a glucose unit, the C_4_-OH group, which is also known as a non-reducing end, the C_1_-OH group, which is also known as the terminating end, and lastly, a reducing end, which is made up of aldehydes. The fibrous structure of cellulose is responsible for its physicochemical characteristics [3]. This structure enables cellulose to be modified, in order to be inculcated in medical use, without losing its native properties. The structure of cellulose is composed of glucose monomers in the beta form, which are attached together through condensation by the glycosidic bond. Cellulose can be classified as α, β, and γ, based on its ability to dissolve in a sodium hydroxide (NaOH) solution, with an optimum concentration [4]. Cellulose is a non-soluble compound, due to the presence of intermolecular and intramolecular forces that exist between the hydrogen bond. The extensive modification to its chemical structure has been performed frequently, in order to improve the capabilities of the cellulosic materials that are used in industry [5], and more recently, in pharmaceutical needs.

The cellulose structure consists of three hydroxyl groups, which exist with repeating units. The hydrogen bond that holds cellulose together has a great influence on the physical characteristics of cellulose. During the formation of cellulose, both Van der Waals forces and intermolecular forces exist between the oxygen and hydroxyl groups. This, in return, induces the aggregation of the cellulose chain that forms the fibrils. Both of these forces make cellulose a stable polymer, by promoting the fibrils with greater axial stiffness [7,8].

**Figure 1:**
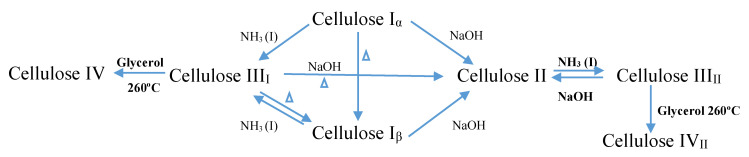
Schematic diagram of polymorph synthesize of cellulose.

Native cellulose exists in two different forms of crystalline, namely cellulose type I and cellulose type II [5], yet generally, cellulose can be classified into four different polymorphs [9]. Cellulose I consists of two sub allomorphs, known as Iα (triclinic) and Iβ (monoclinic) [5,7,10]. Cellulose type I and cellulose type II happen in both parallel and antiparallel axes, due to the presence of the intra- and inter-molecular forces that exist in the microfibrils of cellulose type I [8]. Cellulose type I is naturally synthesized in plants, in bacteria, and in algae, and by dissolving cellulose type I in an aqueous NaOH solution, cellulose type II can be produced. Cellulose type III is produced by treating cellulose type I, or cellulose type II, with liquid ammonia, while cellulose type IV is produced through the thermal procedure [5,8,11]. Among these types, cellulose type I is less stable thermodynamically when compared to cellulose type II, which is the type that is stable in nature [5,8]. Chemical formula of cellulose as in Figure 1.

## 4. Plant Cellulose

Cellulose is a natural organic compound that is present in plants and it makes up a major component of the cell wall in a plant. Cellulose can be obtained from various sources. Generally, plants serve as the main source of cellulose. The cellulose that is extracted from plants exhibits an exceptional property, which makes it suitable to be widely used in the pharmaceutical industry. Cotton and woods are main sources of production of cellulose.

For instance, kenaf, which is also known as *Hibiscus Cannabinus,* is one of the most widely used plants to extract cellulose, due to its mechanical strength [12]. Plant-derived cellulose is naturally stable and non-toxic. It has been speculated that the type of cellulose that usually appears pure, is particularly from the hairs of cotton. Cellulose from wood usually contains lignin and other substances (polysaccharides). These must be extracted by using a chemical pulp, and then through the purification procedure, prior to their use as cellulose. Apart from this, cellulose can be extracted from bamboo and rice husk. Cellulose can be modified to form hydroxyethyl cellulose, hydroxypropyl methylcellulose, and sodium carboxymethyl cellulose, which can then be utilized in the pharmaceutical industry [3]. Plant cellulose is synthesized through rosette terminal complexes, which are comprised of cellulose synthase and related enzymes. Cellulose synthase will be primarily isolated from plots of hydropathy. Its amino acid structure is composed of eight transmembrane segments. The eight transmembrane segments are joined by several longer loops that are attributed to the cytosol and a vast array of short loops on the transmembrane’s exterior. The formation of a new cellulose chain starts as a lipid membrane, which involves a transition of UDP-glucose from glucose to sitosterol. The process is followed by adding more glucose residues to the initial β 1–4 linkage. As a result, a short chain of oligosaccharides will be attached to sitosterol, forming a dextrin of sitosterol, which then flips across to the external surface of the plasma membrane. Here, the polysaccharide chains will be removed with Endo-1, 4-β-glucanase [13]. The removed dextrin primer now binds covalently to the other portion of cellulose synthase. UDP-associated glucose is α-linked. Glycosyltransferases will convert its configuration so that the product (cellulose) is now β-linked [8,14].

## 5. Bacterial Cellulose

**Figure 2:**
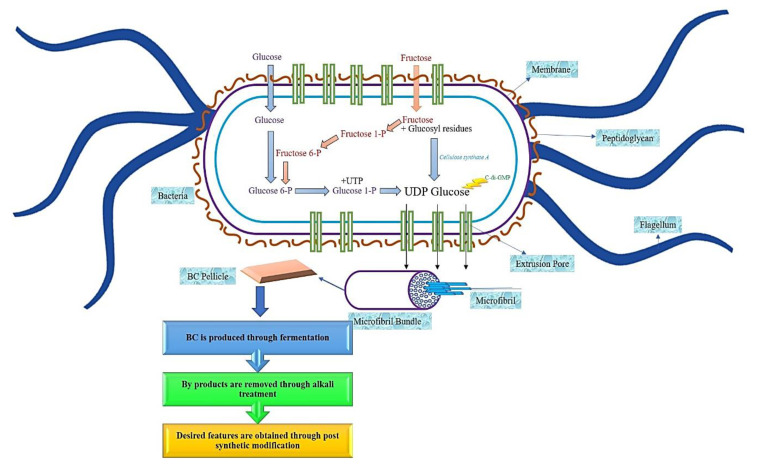
Schematic diagram of bacterial cellulose synthesize.

Bacterial cellulose (BC) can also be derived through the fermentation process [2], specifically, if the bacteria are aerobic bacteria [4,15]. BC is usually synthesized from bacteria that belong to the genera *Gluconacetobacter* [9], *Agrobacterium*, and *Sarcina*, through an oxidative fermentation process or by microbial fermentation [11,13], and when modified, BC will form a substance resembling a cartilage [16,17]. In contrast, aerobic Gram-negative bacteria belong to the genus *Gluconacetobacter hansenii*, and it has been proven to support and produce a greater amount of cellulose when cultured in a sugar-rich liquid medium [18,19,20]. The production of BC comprises of the same stages as PC, which is through the polymerization of the glucose units to produce the β1→4 glucan chain, followed by the crystallization of the cellulose lineage. This procedure comprises of four main stages, namely, the phosphorylation of glucose to glucose-6-phosphate by glucokinase, followed by the isomerization of glucose-6-phosphate to glucose-1-phosphate by phosphoglucomutase. Then the transition of glucose-1-phosphate to uridine diphosphate glucose (UDP-glucose) by UDP-glucose pyrophosphorylase, preceded with cellulose synthesis by UDP-glucose synthase. Due to the utilization of disaccharides as the source of carbon for BC synthesize, the process of BC biosynthesize begins with the hydrolysis of disaccharides into monosaccharides, such as glucose and fructose [16]. A linear chain of glucose will be produced in the body of the bacteria during the synthesis process, as shown in Figure 2. Through tiny pores that exist in the body of the bacteria, the glucose will be squeezed out. This glucose will come together to form the microfibrils.

**Figure 3:**
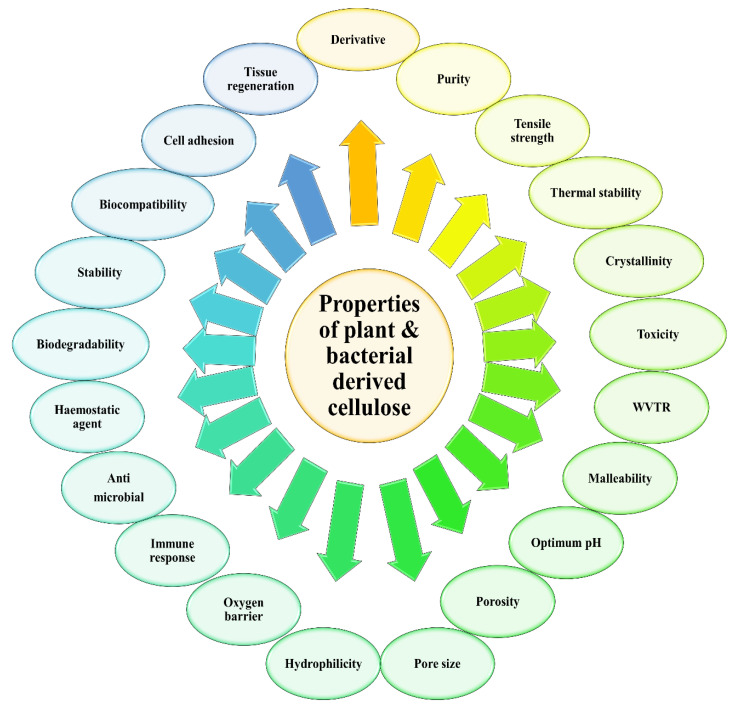
Related properties to be compared for plant and bacterial derived cellulose.

Over time, the accumulation of these microfibrils will lead to the formation of the cellulose ribbon. A web-shaped interconnected pattern will be produced by this cellulose ribbon, with a sufficient amount of empty spaces existing between the fibers, which creates a highly microporous structure [21,22]. BC demonstrates a well-distributed 3D structure of nanofibers, with a large surface area, a high tensile strength, and with a water-absorbing capacity [11]. The structure of the BC end-product is similar to PC, which is encompassed by a glucan chain with β-1→4, and held together by the hydrogen bond, with a molecular formula of (C_6_H_10_O_5_) [17]. The polymerization occurs by adding residues of glucose to the non-reducing end, or to the lipid intermediate involvement. After the fermentation process, BC appears impure, due to the presence of cells and their medium composition. In order to purify BC, the pellicles are extracted from the fermented broth, followed by a mixture-washing using purified water. To dispose of the BC cells, the solution is then combined with the organic acids, potassium hydroxide and sodium hydroxide. An aspirator is used to filter the solution, and the filtrate is washed until BC is obtained with a neutral pH [16]. The comparison of BC and PC properties has been summarized in Figure 3.

## 6. Enzymes as a Protection Agent for Bacterial Cellulose

The usage of BC as a wound dressing material, specifically for chronic wound is common. Although BC does not naturally have intrinsic antimicrobial property, it can be easily integrated with certain elements to exhibit antimicrobial protection. Unfortunately, the efficacy of pre-saturated BC can be hindered due to the presence of biofilm produced by certain bacteria. To cater for this issue, researchers have studied a few methods that can be effective against prevention of biofilm protection. This includes immobilization of lysozyme and glycoside hydrolase in BC [23,24]. In this context, several studies suggested that integration of lysozyme may increase the size of the particles which may cause significant alteration in specific activity [25]. However, correct optimization of lysozyme in BC has proven to be effective in inhibiting the formation of biofilm. In this scenario, Bayazidi and co-workers (2018), demonstrated that 31.18 µg/mL immobilization of lysozyme with a pH of 7 in BC demonstrated a notable effect of antimicrobial activity against *Escherichia coli, Yersinia entrocolitica, Staphylococcus aureus, Aspergillus niger, Listeria monocytogenes,* and *Saccharomyces serviseae* [26]. Meantime, integration 32.35 ± 1.05 mg glycoside hydrolase in BC proven to be effective in reducing elements of biofilm’s polysaccharide in BC. At the same time, they showed rapid destabilization of biofilm produced by *Pseudomonas aeruginosa*. Similarly, absence of cytotoxic effects towards L929 fibroblast cells further indicated that glycoside hydrolase is an alternative choice for preventing biofilm mediated infection in wound dressing [23].

## 7. Characteristics of PC and BC

The physicochemical properties of plant cellulose (PC) or bacterial cellulose (BC) greatly influence the overall characteristics of the cellulose end-product. This will include all of the types of cellulose that are being modified for the industrial and pharmaceutical processes. As a result, PC and BC are widely being inculcated for therapeutically approaches, due to their unique properties in accelerating the wound healing phases. The characteristics of cellulose are extensively described in Table 1.

### 7.1. Thermal Stability

Thermal stability is characterized by the ability of the polymer to withstand temperature, without affecting its features, such as elasticity, strength, and so forth [52]. An optimum range of thermal stability is needed to enhance the epidermal migration and blood flow to the wound bed in an injury [51]. The thermal stability of regenerated cellulose (RC), which is produced from cotton linter fibers, has been broadly studied, and it has exhibited greater thermal stability, with an initial decomposition temperature of 299 °C, a maximum decomposition temperature of 328 °C, and final decomposition temperature of 345 °C [29]. This proves that RC possesses a moderate crystallinity (40–60%) index [30], with a huge crystalline size, in accordance with the experiment that was executed by Poletto et al. (2012). They noticed that the crystalline size and index influenced the thermal degradation temperature of the cellulose end-product [76]. These findings have been supported by Santmarti et al. (2018), when they unraveled that the cellulose chains were orderly arranged and densely packed at high temperatures. Thus, this indicated a high degree of crystallinity. This particular phenomenon is the main challenge of major factors that hinder cellulose degradation at high temperatures. In contrast, less stability of a cellulose structure is commonly found in the amorphous phase, especially when it can be observed during the pyrolysis of cellulose [77]. Hence, this clearly shows that cellulose crystallinity totally affects the thermal stability properties. Meanwhile, BC, when it was prepared from nata de coco, presented a glass transition temperature of 191 °C, with an absence of degradation in between the temperatures ranging from 0 °C–250 °C. This indicated a greater thermal stability that was attributed to the presence of the fibril structures and crystallinity (84–89%), together with an absence of impurities [16,27,28,29].

### 7.2. Hemostatic Effect

Hemostasis refers to a state of bleeding discontinuation while preserving the blood fluid level and the removal of the clot upon the vascular integrity restoration [78]. This is one of the properties that is needed in any wound dressing, in order to stop the bleeding at the injury site. In accordance with this, plant-derived sodium carboxymethyl cellulose (SCM-PC) exhibits a hemostatic effect, through the action of fibrin polymerization, without stimulating the blood coagulating factors. SCM-PC induces the formation of the fibrin fibers, which activate plasminogen and cause the plasminogen activator to bind to the fibrin. This increases aggregation of the fibrin monomers, by means of the two-stranded fibrin protofibril formation [59]. Apart from this oxidized regenerated cellulose (ORC), due to the presence of the fibers within it, ORC serves as a platform for an aggregation of the platelets [60]. This, in turn, controls the diffused bleeding in a wider area [63]. Furthermore, regenerated cotton cellulose (RCC), a plant-derived cellulose, has the ability to absorb blood at the injury site. This may assist in the blood coagulation process, by creating a conducive microenvironment for the wound healing [61]. Moreover, by oxidizing the BC membrane through tetramethylpiperidine-1-oxyl, this has been proven to exhibit a hemostatic effect [58].

### 7.3. Biocompatibility

Biocompatibility refers to the reaction of the biological tissue towards foreign or implanted material. In this review, plant (PC) and bacterial (BC) cellulose have been discussed in detail. Biocompatibility is vital, as this influences the adhesion, proliferation, and the survival of the cells, upon the implantation of cellulose at the wound area. For instance, PC, when it is derived from the hypanthium tissues of apples, enhances the cell infiltration, comprising of eosinophils, polymorphonuclear cells, and granulocytes, which surround the implanted-cellulose, with an absence of dead tissues [65]. The presence of the microporous structure of PC, in which the range of distribution is between 73 to 288 μm [75], enhances the formation of new blood vessels [65].

The biocompatibility of BC is proportional to its ability to absorb water, in order to assist growth and cell proliferation [79]. A moderate to a high level of biocompatibility of BC has been identified, due to its ability to support living cells for up to 12 weeks in the subcutaneous tissue [80]. In this scenario, a complete absence of exudation, giant cells, and inflammatory responses, such as redness and swelling appearances, have been witnessed [80]. BC hydrogel that has been modified with an extracellular matrix, such as hyaluronan, collagen (Col), or elastin and growth factors (GF), including keratinocyte growth factor (KGF), human basic fibroblast growth factor (B-FGF), and human epidermal growth factor (H-EGF), has been able to inculcate these biocompatibility characteristics into BC. This strategy has resulted in an improved biocompatibility rate and it has provided a positive effect as a skin substitute [81]. In addition, pure BC possesses natural characteristics as a compatible agent to blood, whereas modification to the internal structure and the porous microstructure, further induces the ingrowth of cells [30]. This property enhances the adherence of the smooth muscle cells, chondrocytes, and endothelial cells to BC [71].

### 7.4. Degradation Property

Plant cellulose (PC) is difficult to degrade when compared to bacterial cellulose (BC). The fact is that PC presents a ribbon structure in a complicated manner when compared to BC and moreover, PC is composed of impurities, such as pectin, hemicellulose, and lignin [42,43]. Meanwhile, a compact fibrous microstructure and a high degree of crystallinity contribute to the slow degradation of BC and this exhibits a less amorphous phase. Yet, BC is easily degraded when treated with an acid solution. On the contrary, a longer duration is needed to hydrolyze PC with an alkali solution (15% NaOH), in order to achieve a full degradation [62]. On top of this, periodate oxidation simulates the body fluid, and phosphate buffered saline (PBS) has the potential to degrade BC, without distracting the existing fibrous structure [79]. The study that was performed by Portela et al. (2019) described that BC, when integrated with chitosan, can be degraded by the lysosomes that are present in the human body, whereupon this released the mono and oligosaccharides upon the degradation. As a result, this stimulated the production of new blood vessels, which led to the rapid regeneration of the tissues [35]. Therefore, this degradation property intuitively affects the angiogenesis process, which plays a major role in expediting the wound healing process.

### 7.5. Antimicrobial Property

Protection against foreign bodies, such as bacteria, is essential for rapid wound healing, without any delay. An open wound is highly susceptible to a bacterial infection and this hinders the normal mechanisms of wound healing. The presence of bacteria at the wound site will induce the release of a high accumulation of toxic substances [82], such as endotoxin and exotoxin [83]. Over time, the accumulation of toxin will hinder the capacity of the immune cells to combat bacteria and this will lead to the formation of biofilms at the wound area. The formation of biofilms slowly grows and further spreads, with a surrounding of the wound site, and this complicates the process of wound healing [82]. Generally, BC in a native form does not exhibit any antimicrobial effects. When considering this, bacterial cellulose (BC) can be used for wound healing, due to its antimicrobial properties, with a combination of antibacterial compounds and nanoparticles. However, when BC is incorporated with silver (Ag) nanoparticles, this serves as an effective antimicrobial agent towards *E. coli*, with a reduction of 99.2% of the bacterial colony that was previously observed [84]. These silver nanoparticles (AgNP) anchor the walls of the microbes through an electrostatic force and this causes infiltration of AgNP and damages the membrane of the microbes. Eventually, the interaction of AgNP with the DNA of bacteria leads to dysfunction, finally causing leakage of the content, leading to cell death. Through this method, the highest inoculation of antimicrobial activity has been witnessed against *Pseudomonas aeruginosa (P. aeruginosa), Escherichia coli (E. coli),* and *Staphylococcus aureus. (S. aureus)*. The minimum inhibitory concentration (MIC) of 200, 600, and 800 μg/mL against the abovementioned microbes have been identified with a release of 0.33, 0.08, and 0.29 μg/mL of AgNP, respectively [85].

Nevertheless, the incorporation of fusidic acid, tetracycline, amoxicillin, erythromycin, povidone-iodine, octenidine dihydrochloride, polyhexanide, benzalkonium chloride, laccase, and quaternary ammonium compounds with BC, has been scientifically proven in combating the microbes [35]. In addition, BC naturally has the capacity to hinder the penetration of bacteria into the tissue, by acting as a physical barrier [35]. Previously, BC was incorporated with polylactic acid (PLA) and this seemed effective in inhibiting the bacteria invasion, specifically against *E. coli* and *S. aureus* [86]. Apart from this, by reinforcing BC with a dehydrogenative polymer of coniferyl alcohol, AgNP, zinc oxide, gold nanoparticles, and graphene oxide, or with silver nanohybrid and chitosan, this has also been proven to combat bacteria [35]. Thus, BC, when further reinforced with antibacterial components, has a high potential to kill bacteria in a controlled manner, while preserving the moist microenvironment, so as to expedite the wound healing progress.

### 7.6. Mechanical Strength

Young’s modulus and tensile strength were extensively extracted during the evaluation of the mechanical strength for PC. The data showed that the range of Young’s modulus appeared within 120–140 GPa, while the tensile strength was within the range of 750–1080 Mpa, and this was for PC with a density of 1450–1590 kg m^−23^. This was mainly due to the structured anisotropy arrangement of the PC microfibrils, in terms of the mechanical strength [27]. Meanwhile, BC exhibited Young’s modulus within the range of 15–35 GPa [30], with a tensile strength of 146 MPa, 7.4% of elongation capacity, 454.6 MPa modulus of elasticity, for BC with a thickness of 100 µm. The mechanical strength of BC appeared to be tough, firm, and extendable, which indicated a maximum breaking strength [19], with a moderate elongation resembling the features of an ideal bioscaffold feature [28].

### 7.7. Oxygen Barrier

Oxygen is an essential element for wound healing, and yet, the deprivation of oxygen to the tissue does trigger angiogenesis. This hypoxic condition triggers neovascularization (formation of the blood vessel) at the wound site [87]. It might possible due to the hypoxic state, the vascular beds will start to expand, with the presence of the angiogenic factors, such as VEGF at the wound bed [88]. The vascular formation will invade the clot and be organised into a microvascular network of granulation tissue, thereby accelerating the wound healing mechanisms [89]. Hence, it is important to ensure that the oxygen gradient and cellulose play main roles at the wound healing site, in order to provide such properties as the nanocellulose forms. Nanocellulose, a plant-derived cellulose, reflects a strong oxygen barrier to the compact structure as formed by the nanofibrils, with smaller and more consistent dimensions upon its fabrication modalities. When considering this aspect, the pores that exist inside the nanocellulose serve as a key route for the oxygen molecules to be permeated [36]. Meanwhile, a hybrid of BC with other materials, such as with PLA, has been scrutinized to be an excellent oxygen barrier at a relative humidity of 70% [54,90].

### 7.8. Water Vapour Transmission Rate

An ideal moisture surface is essential for the wound healing mechanism because a moist environment at the wound site prevents dehydration. The absence of dehydration favors angiogenesis and Col production at the injury site, followed by the breakdown of dead tissue, and the fibrins will eventually be reduced [91]. As a result, the cycle of wound healing continues. An ideal range of water vapor transmission rate (WVTR) is around 2000–2500 gm^−2^ day^−1^. This hinders the dehydration of the wound, by retaining adequate moisture at the injury site [35]. According to the study that was conducted by Nair et al. (2014), PC has a high WVTR when compared to BC. Furthermore, the main factors affecting this phenomenon include the size and limited swelling capacity, especially nanocellulose, which has a significant reducing effect on the diffusion of the water vapor [36]. Meanwhile, BC shows a water-holding capability that ranges from 60 to 700 times in the hydrated state, based on the process of synthesizing [35]. In addition, this gives BC a native hydrophilicity property and a microporous structure that exists in the wet pellicle [92]. Thus, in return, this gives rise to the interstitial space of the internal surface, as a matrix that never dries out [30]. This directly provides a positive environment to accelerate the wound healing phases.

### 7.9. Immune Response

The post-implantation of PC has been correlated to the immune response activation in the subcutaneous tissue, which is eventually resolved within four weeks [37,43]. In contrast, a high crystalline property has been proven to not be directly correlated with the immune response [53]. This has been scrutinized, as the interaction of nano-based cellulose with the cells did not trigger any presence of the inflammatory mediators [33]. This was because the dendritic cell, which is also known as an antigen-presenting cell (APC) in the human body, acts as a messenger between the innate and immune response. The APC will recognize nano-based cellulose and it stimulates a tolerance to the immune through the expression of the inhibitory molecules, including indolamine dioxygenase (IDO)-1, immunoglobulin-like transcript (ILT) 3, and anti-inflammatory cytokine IL-10, thereby stimulating the anti-inflammatory response [93]. In fact, since PC possesses a high degree of crystallinity, a limited immunologic response can be expected. Meanwhile, a post-implantation of BC in the subcutaneous triggers a mild inflammatory response from the seventh until the 30th day, which eventually resolves on its own. Additionally, the new blood vessel formation inside and surrounding BC will have witnessed an absence of inflammation signs. Thus, in return, this triggers the fibroblast infiltration at the region of BC, which is implanted [61,62], and it expedites the proliferative phase in wound healing.

### 7.10. Cytotoxicity and Cell Viability

The interaction of PC with human cells has shown no significant cytotoxic effects [33], after being tested in a cell culture model of co-cultured lung cells [34]. BC showed no cytotoxic effects upon the seeded MDA-MB-231 cells onto the fabricated BC. Instead, there was a notable rapid rate of cell proliferation for up to three days [31], and the rate of BC cytotoxicity has been graded within 0–1 [32]. This was because pure BC showed a relative growth rate of more than 70% for all of the seeded cells (i.e., the L929 fibroblast and osteoblast cells) [32]. Similarly, PC has been found to enhance rapid growth and cell proliferation. The PC structure deems to attract the seeded cells, with a varying morphological structure, through an adherence on the PC surface, and it grows consistently within the three-dimensional microenvironment of cellulose. This indicates that PC induces a high degree of cell viability, with an increased density of cells. PC has a porous microenvironment, in which nutrients are being transferred to the tissue, in order to enhance the growth of cells in a cellulose bioscaffold [74]. In addition, BC has demonstrated an almost a similar property to PC, in terms of cell viability support. Previously, the implantation of BC when it was seeded with the co-cultured fibroblasts and chondrocytes presented a good integration in in vivo testing. Thus, this has indicated that BC supports and promotes cell attachment and proliferation. These findings were supported by the assessment of an in vitro model, which proved that 95% of the cell aggregation in BC was still alive. In addition, a modification of BC with nitrogen plasma increased the cell affinity to BC, by increasing the porosity of BC [66]. Therefore, it is essential to induce the viability of the cells in BC, which assist in the acceleration of wound healing.

## 8. Mechanism of Wound Healing

Following an injury, bleeding is an immediate response in initiating blood clotting cascade. The mechanism of coagulation, and vasoconstriction starts with rapid impregnation of the wound by clotted blood, leading to hemostasis. It is accompanied by an influx of inflammatory cells, releasing cellular mediators into the injury site. This process is preceded by formation of blood vessel (angiogenesis) and re-epithelialization, in which new cellular and extracellular components will be deposited [94]. All this happens in four overlapping phases; hemostasis phase, inflammatory phase, proliferative phase, and remodeling phase.

Injury usually results in the outflow of lymphatic fluid and blood. It is also the phase where the initial reparative coagulum is formed. When this happens, both intrinsic and extrinsic blood clotting cascade will be activated. The extrinsic pathway is enjoined by thrombocytes while the intrinsic pathway by the damaged tissues. Upon vasoconstriction, platelets will bind to the injured endothelium. Adenosine diphosphate (ADP) will be released which facilitates thrombocytes clumping. Upon completion of short-lived vasoconstriction, the blood vessel will start to dilate to allow more influx of thrombocytes enter the injured area. These thrombocytes, together with other blood cells will triggers the activation of other factors to accelerate the healing mechanism such as platelet factor IV, alpha granules liberate platelet derived growth factor (PDGF), and transforming growth factor (TGF-β). A host of cytokines and growth factors mediate the inflammation process, formation of myoblasts from transformed fibroblasts, deposition of Col, angiogenesis and re-epithelialization [95]. Moreover, the formation of blood vessels id greatly influenced by the presence of vascular endothelial growth factor (VEGF) whereas angiogenesis and re-epithelialization is regulated by fibroblast growth factor (FGF)–2. Thrombocytes will release serotonin and histamine (vasoactive amine). At the same time, TGF-β will act as a modulator for fibroblastic mitosis and PDGF as a chemotactic agent for fibroblasts, leading to the formation of Col in the later phase. The framework for completion of the coagulation process is initiated when fibrinogen cleaved into fibrin [96].

The very next phase is the inflammatory phase. Although the inflammatory process starts during the hemostasis period, the early part of the inflammatory phase is predominated by the influx of polymorphonuclear leukocytes (PMNs) and the later monocyte/macrophage prevailing components. The next stage of the healing process is under way within the first 6–8 h, with PMNs engorging the wound. TGF-β facilitates the migration of PMNs from nearby blood vessels, where they extrude from those vessels. Within the next 24–48 h, PMNs will reach maximum rate and this will start to drop from the 72 h. At the same time, certain chemotactic factors such as plasma activated complements, PDGF, FGF, TGF-α, and TGF-β will be released. These will be sequestered in the scab or eschar by macrophages. Monocytes also exude out of the vessels as the cycle progresses. The macrophages resume the cleaning cycle and different GF will be released simultaneously within 3 to 4 days. The macrophages orchestrate the proliferation of endothelial cells via the sprouting of new blood vessels, replication of smooth muscle cells, and formation of the fibroblast-created milieu [94].

The healing mechanism proceeds with the following phase known as proliferative phase. This phase can be further divided into subphases, which are overlapping phases and occurs concurrently. The subphases includes, fibroplasia, matrix deposition, angiogenesis, and re-epithelialization. By day 5, fibroblast will migrate to the injury site to deposit new Col, specifically Col type I and III. Initially, type III Col predominates, to be later replaced by type I Col. Tropocollagen will act as a precursor for all forms to Col processed within the rough endoplasmic reticulum of the cell in which lysine and proline will be hydroxylated. Disulfide bonds will be formed to allow three strands of tropocollagen to form a triple left-hand triple helix known as procollagen. As the procollagen secreted into the extracellular space, peptidases in the terminal peptide chains of the cell wall will cleave, creating true Col fibrils. The wound will be suffused with glycosaminoglycan and fibronectin. During this process, proteoglycan will covalently bind to the protein core, causing the deposition of matrix. Meanwhile, FGF and vascular endothelial GF will modulate formation of new vasculature. Re-epithelization begins with cell migration from the wound periphery to the adnexal structures. This cycle ends with cell replication within 24 h. Peripheral cell division occurs in 48–72 h, resulting in a thin layer of epithelial cells which bridges the wound. It is believed that epidermal GFs play a key role in this aspect of wound healing [97].

The last phase in wound healing is remodeling phase. The wound undergoes constant changes after the third week. This is known as remodeling. Col is degraded and deposited in a balanced manner which results in no change in the amount of Col present in the wound. In normal wound healing the collagen deposition reaches a plateau by the third week after the wound has been formed. Wound contraction is an ongoing mechanism that stems in part from the replication of the advanced fibroblasts called myofibroblasts, which resemble smooth contractile muscle cells. The optimum tensile strength of the wound is attained by the twelve weeks [98].

## 9. Alternative Biomaterials for Wound Healing

Apart from BC and PC, other products such as hydrogels, poly lactic-co-glycolic acid (PLGA), and 3D living constructs are being studied for their effectiveness in wound healing [99,100,101,102]. In current era, 3D bioprinting gaining much attention as other available biomaterial due to its ability to design cell components in microarchitecture. Interestingly, since the cell origin is from patient itself, there is a low possibility for immune rejection. However, the challenge here is the bio-ink [103]. Fortunately, researchers have concluded that hydrogels prepared from hyaluronic acid is a convenient and safe source due to its cell friendly nature. Moreover, within a short period of time, hydrogels can be tailored with methacrylic anhydride and 3,3′-dithiobis (propionylhydrazide) with the presence of UV. This ensures a proper microenvironment for the wound, which in turn accelerates the normal healing mechanism. Other than this, excellent biocompatibility, absorption capacity, rapid degradation rate, and high swelling ratio are some of advantages integrating hydrogel as a choice for wound healing [99].

Apart from this, combination of Col and gelatin methacrylamide doped with tyrosinase has shown promising result as an alternative choice for skin bio-ink. This appears to be in stable modulation when being used for 3D bioprinting. Simultaneously, this modified bio-ink has shown > 90% of cell viability has been recorded for selected cell lines. In the context of wound healing, the designated biomaterial demonstrated that complete wound closure has been achieved by day 7 in the experimental group. Similarly, acceleration in the formation of dermis and epidermis layer is notable. This clearly shows that such modulation is a perfect biomaterial for wound healing [101].

Nevertheless, integration of andrographolide-loaded mesoporous silica nanoparticles (MSN) to PLGA has also proven to be effective in healing wound through in vivo testing. Although several reported study states that MSN is absence of immune response yet integration of MSN into PLGA could be challenging as well, particularly if the PLGA has initially been loaded with other drugs. However, due to the malleability property, it is easy to incorporate MSN to functional surface to regulate the release of drug in response to different stimuli. A study done by Jia et al. demonstrated that the integration of andrographolide-MSN enhanced hydrophilicity, increased mechanical strength, optimum porosity and pH, possessed strong antibacterial properties and enhanced epidermal cell adhesion and growth. This modification thereby stimulates the rapid wound healing in the in vivo testing [100].

## 10. Uniqueness of Bacterial and Plant Cellulose in Comparison with Alternative Biomaterials

Cellulose regardless of its derivative can be categorized as an inexhaustible natural source of polymers that available throughout the world. It stands as an unique and most widely used biomaterial due to its outranges characteristics such as high mechanical strength, malleability, ability to hold water, high degree of crystallinity, presence of 3D-network made of linear b-1, 4-glucan chains, and the list goes on [104]. Nevertheless, with a minimum amount of modification PC and BC have been proven scientifically to be used for skin healing, wound dressings, template for bone tissue, 3D nerve cell proliferation and differentiation, artificial blood vessels, cartilage, urinary tracts, vertebrae disks, larynx tissues, ligaments, cartilage, tendons, muscles, etc. [67]. Interesting, absence of immune reaction toward cellulose has been discovered which further makes the cellulose as a better choice compared to other available biomaterials [105]. Nonetheless, cellulose has been proven to have very high surface area per unit mass which maximize the outcome upon application. Thus, cellulose serves as an attractive candidate for wound healing application [106].

## 11. Plant Cellulose for Wound Healing

The use of cellulose in therapeutics has no boundary, and plant cellulose (PC) is not excluded in this era, as shown in Table 2. Previous experimental studies have proven that PC is suitable to be used for wound healing, due to its unique physicochemical properties. Singla et al. (2015) conducted a study on *Syzygium cumini* cellulose in diabetic wound healing, with full removal of the PC impurities. The incorporation of PC with AgNPs to inculcate the antimicrobial property demonstrated a non-toxic effect towards the in vitro cytocompatibility assessment. This was because 60–70% of the cell viability that was revealed in the keratinocytes, even after 48 h, was after being applied as a topical dressing to diabetic-induced Swiss albino mice.

The size of the wound closure was further observed on days 3, 10, and 18. The initial assessment (on day 3) showed a slight wound closure, with an absence of infection and with evidence of a smaller ratio of the neutrophilic granulocytes. On day 10, the wound evaluation was described as a complete re-epithelization, with the loss of a scab when compared to the control group. In addition, a well-structured dense bundle of Col and migrated fibroblasts were seen. Finally, on day 18, the wound evaluation presented evidence of an efficient process of wound healing, with a reconstitution of the dermal-epidermal junction (DEJ). Thus, the results that were obtained in this study have shown that modified PC promotes rapid wound healing. This is possibly due to the incorporation of AgNPs into PC, as it was proved to exhibit anti-inflammatory properties [109], with an inhibition of the cytokine and macrophage infiltration. This, in return, decreased the duration of the granulated-tissue formation, resulting in fast wound healing [107].

Modulevsky et al. (2016) through their in vivo experimental study proved that cellulose, when derived from apple hypanthium, could be used in tissue engineering. This study evaluated the compatibility of decellularized plant-derived cellulose in terms of cell infiltration, extracellular matrix deposition, and vascularization, upon implanting a cellulose scaffold in an artificially incised wound of mice. The evaluation at the first week showed a moderate level of an immune response, with infiltration of the polymorphonuclear (PMN) leukocytes, eosinophils, and dead cells, surrounding the implanted cellulose scaffold. The immune response was eventually resolved on the fourth week, as only a high level of lymphocytes and macrophages were seen around the scaffold. This showed an immediate immune reaction of the native tissue towards the scaffold, which was recognized as a foreign material upon implantation as a cleaning mechanism, as the immune response eventually resolved on its own. The assessment at the eighth week showed an active migration of the multinucleated cells, macrophages, and fibroblasts towards the scaffold, indicating the possible formation of a new extracellular matrix and a deposition of Col. At the same time, there was an increased formation of blood vessels and capillaries visible within the scaffold on the fourth and eighth week. The overall obtained results in the Modulevsky et al. (2016) study has indicated that plant-derived cellulose is biocompatible to human tissue, as there were not any notable signs of inflammation [65]. This has shown that PC has the capabilities to support tissue regeneration, as it displayed natural pro-angiogenic characteristics [110].

Modulevsky and co-workers (2014) previously described the efficiency of apple derived cellulose (AC) when it was used in mammalian cells. The McIntosh red apples were decellularized and chemically cross-linked to attach the rat’s tails to the Col. Cellulose was cross-linked with hemicellulose, which actually mimics the extracellular matrix [74]. The fabricated AC bioscaffold was seeded with three types of cells, C2C12 myoblasts, NIH3T3 fibroblasts, and HeLa human epithelial cells. Cell proliferation was identified as increasing by 3- to 4-fold in the 12th week, most probably because AC consisted of a microporous network that facilitated the transfer of nutrients. The continuous culture of the cells in the AC allowed for the cells to invade and proliferate rapidly. AC supported the viability of the fibroblasts that speculate of the ability to bring in more growth factors to the wound site. Thus, the authors can summarize that AC provides a 3D conducive environment that resembles the extracellular matrix, which enhances rapid cell attachment and proliferation.

A retrospective cohort study that was performed by Masci et al. (2018) proved that oxidized regenerated cellulose (ORC) has the capability to stop bleeding, which was currently impossible with the conventional hemostatic procedure. In this particular study, ORC gauze was placed directly at the liver bed in 24 patients who had recently undergone laparoscopic cholecystectomy surgery. Upon application of the ORC gauze, bleeding discontinuation was observed within three to seven minutes [108]. This scenario was supported by Aoshima et al. (2012) who witnessed a hemostatic effect of a soluble fraction from plant-derived sodium carboxymethyl (Sol-CM) cellulose in an in vivo testing. The findings unraveled that Sol-CM absorbed thrombin and it participated in the formation of fibrin fibers [59]. In accordance with the normal wound healing pathway, fibrin plays a major role in the immediate retraction of the clot. The presence of fibrin further connects more platelets together to be aggregated at the wound site, thus forming a clot [111], and eventually causes the bleeding to stop at the wound region. Hence, ORC has been proven to naturally possess hemostatic characteristics [112,113].

## 12. Bacterial Cellulose for Wound Healing

Bacterial cellulose (BC) is naturally pure, with an absence of lignin, hemicellulose, and other contaminants when compared to PC, and it resembles the extracellular matrix. These special characteristics are the main reasons for the extensive use of BC in biomedical and clinical settings than those of PC. The positive outcomes of BC usage in wound healing have been confirmed through in vivo and in vitro testing [66,67,68,69,70], as described in Table 3. For instance, Wen et al. (2015) have shown the efficiency of BC when incorporated with silver sulfadiazine in wound healing, by using a Wistar rat model with a partial thickness of a burn wound [114]. The results showed the formation of a scab, a partially healed wound, and complete healing (>92%), as observed on day 7, 10, and 14, respectively.

A greater deposition of Col formation was noticeable, showing the progress of the remodeling phase. At the same time, a positive bacteriostatic effect (>99%) towards *E. coli, S. aureus,* and *P. aeruginosa* was seen, where the bacterial count was drastically reduced by day 7, with it less than 103 CFU/cm^2^. The results obtained have indicated that by impregnating silver sulfadiazine to BC, this provides an antimicrobial property [114]. The bacterial invasion through an open wound finally tends to induce an immune response, while it invades and damages the tissue at the wound site. This results in a slow healing process, due to the interference of the microbes with inflammatory cells. This fabricated BC, when incorporated with silver sulfadiazine, prevents the bacteria from entering the wound region, and it continuously accelerates the wound healing, due to the absence of inflammation.

The effectiveness of native BC and BC when incorporated with vaccarin, a drug used in wound healing, has been investigated by Qiu et al. (2016). The results described excellent biocompatibility of the L929 cells in the vaccarin-integrated BC, with more than 80% of cell viability, with a growth rate > 74%. Furthermore, an in vivo study has shown a progressive wound closure on day 7, followed by complete healing on day 14. Both of these days demonstrated a low presence of inflammatory cells and a high number of fibroblasts. The findings strongly support the fact that vaccarin promotes proliferation of the endothelial cells and that it was capable of providing a dose-dependent protective barrier towards the reduction in cell viability [115]. Particularly on the 14th day, both the BC and the vaccarin-integrated BC dressings displayed the formation of Col fibers, subcutaneous tissue, hyperplastic fibrous connective tissue, and the appearance of stratified squamous epithelium at the injury site [116]. BC, when integrated with vaccarin, was more effective when compared to native BC. This supports cell proliferation, specifically the fibroblasts and the granulation tissue, leading to a rapid healing mechanism.

A similar study that was scrutinized by Wu et al. (2014), was to test the efficacy of BC when integrated with silver nanoparticles (AgNP), in a protective mechanism during wound healing. Briefly, around 2.62 wt% (*w/w*) of AgNP was integrated with BC through an electrostatic force. An interconnected porous structure was created on the surface of BC, which was expected to allow for the distribution of AgNP into BC [117]. An inhibition zone of 3.46 mm was visible against *S. aureus*, within a duration of 24 h. Apart from that, an in vivo study has also indicated that the epidermal cell proliferation started from day 4, upon implanting the scaffold in a rat model. This was confirmed by observing the orbicular transparent cells that were obtained from the rats under an optical microscope. The biofunctionalization of BC and AgNP acted as a moisture microenvironment provider and as a protective barrier against the microbes, respectively. These essential factors play main roles in expeditious wound healing, especially in the proliferative and re-epithelialization phase. The migration of keratinocytes under the scab towards the injury site is much easier to achieve on moist surfaces, in order to accomplish the re-epithelialization. This study showed a notable migration of fibroblasts at the wound site at a rate of 0.5 mm/day under the moist conditions when compared with the dry wound site (2 × faster) in a rat model [117]. The healing progress on day 14 promoted a wound closure, with more than 85% and 44% for BC and control, respectively. The release of Ag+ from AgNP significantly reduced an increase of Ag+ concentration in a culture media in vitro. In addition, AgNP is generally non-toxic, due to its ability to control the release of electrons [119] in vivo. The hybridization of AgNP into BC is a safe and effective way to accelerate wound healing.

Kim et al. (2013) demonstrated the usage of a BC nanofibrillar patch for a wound-healing platform for a tympanic membrane (TM) [118]. The in vitro testing indicated a greater attachment of TM towards the patch and it increased the fibroblast and keratinocyte proliferation. The patch was placed in a mouse model, with an artificially created TM. This resulted in 90% TM regeneration on day 10, with an average threshold of 14.5 ± 1.5 dB for the auditory. The BC nanofibrillar patch contributed to the wound healing, as well as to restoring the normal function of promoting spontaneous healing in all three layers of the TM. TM contained epidermal, connective, and mucosal layers when compared to control, which showed irregular healing in all three layers [118]. From this study, it can be concluded that the nanostructured surface of the BC nanofibrillar patch enabled the adherence of cells to the patch. This spontaneously enhanced the rapid proliferation of cells in all three layers.

Furthermore, an experimental study by Fu et al. (2012) was conducted for skin tissue repair by using BC. In this study, BC was synthesized from *Gluconacetobacter xylinus* and it was modified until the BC film was produced. This BC film measured about 60 × 10 mm and it was then placed into the artificially created wound site, which measured about 10 × 10 mm in a BALB/c mouse. The greater tensile strength of the BC film correlated with the excellent 3D structure of the BC nanofibers. This indicated the similarity of elongation of the BC film, in comparison with the mice. Nevertheless, the similarity of the skin thickness of the mice and the BC film further eased the experiment. The results showed a rapid reduction of the wound region in a mouse that was treated with 2 mm BC when compared to control, which was treated with a thin BC film. At the same time, the water content of BC, which was 98–99%, enhanced the healing by providing a moist environment for the proliferation of cells and the regeneration of tissue. The concentration of TGF-β1 and bFGF was approximately 133.9 pg·mL^−1^ and 1.58 pg·mL^−1^ in the thick film. The increased levels of TGF-β1 and bFGF in the blood contributed to the active proliferation of the fibroblasts [113]. This indicated that a BC film could influence TGF-β1. Generally, TGF-β1 hinders the action of the hematopoietic, endothelial, and epithelial cells. Once the activity of the cells is inhibited, the growth of the mesenchymal cells, such as the fibroblasts, will become stimulated. When this happens, bFGF will exhibit a mitogenic effect on the fibroblasts. This contributes to the production of a new blood vessel and this was attributed to the rapid wound healing of the mice [120].

BC-based dressings that have been approved and marketed include Biofill^®^ (BioFill Produtos Bioetecnologicos, Curitiba, Brazil), XCell^®^ (Xylos Corporation, Langhorne, PA, USA), BioProcess^®^ (BioFill Produtos Bioetecnologicos, Curitiba, Brazil) [75,76], and Gengiflex^®^ (BioFill Produtos Bioetecnologicos, Curitiba, Brazil) [121]. Biofill^®^ was the first commercially approved BC product. It functions as a temporary skin substitute, which can also be used as wound coverage [122]. A BC dressing has to meet the demands for an ideal wound dressing. Moreover, Biofill^®^ can perfectly match the wound site, as it is durable, elastic, permeable to water vapor, easy to use, and it acts as a protective barrier against the microbes, whilst exhibiting hemostatic properties. These characteristics enhance the rapid healing of a wound and this has been scientifically accepted and proven in more than 300 cases [121]. XCell^®^ is a commercially available BC dressing that has the capacity to release and absorb water at the wound site. This product assists in wound healing, by controlling the moisture microenvironment, and it conforms equally to damaged and healthy skin [123]. The XCell^®^ product has been proven to be effective in treating chronic skin abnormalities [124]. Meanwhile, the BioProcess^®^ product is widely used to treat burns and ulcers. Since BioProcess^®^ is incorporated with an antibacterial property, this dressing accelerates wound healing, by acting as a protective barrier at the wound site [35]. Gengiflex^®^ is a 2-layer membrane, in which BC has been modified by using an alkaline solution. This product is mainly used in dental implants to treat wounds that are associated with an osseous deficiency. The outcome measures have shown that Gengiflex^®^ contributes to rapid healing of the periodontal tissue, by reducing the inflammatory response [37,78,80].

## 13. Current and Future Prospect of Plant-Derived/BC in Therapeutics

The potential usage of BC is now envisaged in the pharmaceutical and cosmetic industries as follows. BC has been inculcated as (1) an emulsion or hydrogen to be utilized as surfactants in Pickering emulsions. BC has also been modified to (2) immobilize the action of enzymes and biomolecules, in order to improve the effectiveness in an in vivo study and to achieve maximum stability. Furthermore, BC has also been widely used as (3) a drug delivery mechanism, to enhance the uptake of medication by specifically targeted cells. BC has even been used as (4) a diagnostic sensor to anchor translation and immunoglobulins at a minimal cost. Last, but not least, BC has been approved to be used as (5) an artificial skin substitute and as regeneration of tissue for wound healing purposes [125]. Above all, BC provides an important framework in the advancement of high-tech bio-platforms for diagnosing and treating a vast range of diseases.

The introduction of new pharmaceutical products from natural sources for a therapeutic application is vital. This has been always the greatest concern for biologists and material scientists. When considering the positive impact of inculcating natural polymers into biomedicines, about $97 billion has been allocated for the nanocellulose impacted markets in the pharmaceutical and health sciences [126]. This is mainly for nanocellulose based studies as a future perspective in biomedicines. A few organizations have officially announced to demonstrate cellulose nanofibril and cellulose nanocrystal plants in Europe and North America. They mainly concentrate on producing this specified cellulose in their home country on a large scale [126].

## 14. Conclusions

Cellulose is widely accepted to be incorporated into the biomedical field, due to its biocompatibility to human cells. Both PC and BC differ in their means of macromolecular characteristics. PC contains impurities, such as hemicellulose and lignin, with a moderate water holding capacity (60%), and it possesses a moderate level of tensile strength and crystallinity. Meanwhile, BC is chemically pure. It has a hydrophilic and high-water holding capacity (100%), with high crystallinity, and it possesses a high tensile strength. A wide range of studies has been performed on BC and PC, in order for it to be potentially used in wound healing and in therapeutics filed. With regard to this review, BC and PC may act as a scaffolding layer for the recovery of a vast range of cells and tissues, demonstrating that it may ultimately emerge in the future to become an exceptional platform for medicinal technology. If BC can be mass processed efficiently, it can eventually become a crucial biomaterial that can be used as a substitute for currently available wound dressings.

## Figures and Tables

**Table 1:** Comparison of Plant and Bacterial Cellulose.

Characteristics	Bacterial Cellulose	Plant Cellulose
Derivative	Genera *Agrobacterium,* and *Gluconacetobacter, Sarcina* [19]	Cotton, wood, bast fibers, seed fibers, leaf fibers, fruit fibers, stalk fibers, vegetable fibers and skin [5]
Purity	Pure by nature [16]	Impurities available. Presence of lignin, ash, pectin and hemicellulose [19,20]
Tensile strength	200–300 MPa [22]	750–1080 MPa of native PC with a density of 1450–1590 kg·m^−23^ [27]
Thermal stability	Transition = 191 °C and Decomposition = 0 °C − 250 °C [16,27,28,29]	Initial decomposition = 299 °C; maximum decomposition = 328 °C; final decomposition = 345 °C for regenerated PC [29]
Crystallinity	High. 84–89% [17,27,28,29]	Low. 40–60% for native PC [30]
Toxicity	Absence of cytotoxic effect on MDA-MB-231 [31] similarly, the absence of cytotoxic has seen in L929 fibroblast and osteoblast cell with a grading of 0–1 [32]	Slight cytotoxicity on nanocellulose was observed on HEK 293, causing the rupture of the membrane. Similarly, Cytotoxic was reported when 0.25–5 mg/mL cultured on bronchial cells (BEAS 2B) [33] Absence of cytotoxic effects on V79 was seen on nanocrystalline cellulose [34]
Water Vapor Transmission Rate (WVTR)	High WVTR. Hydrated BC biofilm exhibits 2900 gm^−2^ day^−1^ [35]	High WVTR. 234 g/m^2^ day for nanocellulose film thickness with 42 µm [36]
Malleability	High. Due to the large elastic modulus [37,38], can virtually be shaped in any desired form [39]	Low. Arrangement of microfibrils in the mesh of crisscrossed form gives shape to the lignocellulose in the early stage itself [40]
Optimum pH	5.4–6.3. This influences the O_2_ uptake and growth rate [41]	Non-Applicable
Porosity	High. Appears with a uniform distribution of pore size [42]	Low. Due to the fewer and little space between the fibrils of nano fibrillated of native cellulose [43]
Pore size	10 to 300 nm [37]	1 to 100 nm [44]
Hydrophilicity	High, due to the presence of the hydroxyl group with a high density on the surface of BC. Meantime, extensive H_2_ bonding of chains and crystalline structure enhance hydrophobic interactions thereby contributing to amphiphilic characteristics of the BC [45,46,47,48]	Moderate. The free hydroxyl group exists in the amorphous structure of PC, which enhances the H_2_ bond formation; making it harder for fibers of cellulose to dissolve in water. As a result, only swelling of the fibers occurs. This moisture persists in the H_2_ bond making it less hydrophilic on cotton cellulose [21]
Oxygen barrier	Strong. Addition of PLA to the BC act as excellent O_2_ barrier up to 70% of relative humidity [49,50]	Strong. Due to the presence of small and consistent dimension of nanofibrils on nanocellulose [36]
Immune response	Mild resolves on its own by a maximum of 30th day with an absence of inflammatory signs [51,52]	Mild, which resolves on its own over time. Immune tolerance due to the presence of high crystallinity on native PC [33,53]
Antimicrobial	Absent in native BC. However the integration of BC with AgNP shows effective antimicrobial agents against *Escherichia coli* [54], *Staphylococcus aureus* [55] and *Pseudomonas aeruginosa* [56]. Apart from this, incorporation of BC with fusidic acid, tetracycline, amoxicillin, erythromycin, povidone-iodine, octenidine dihydrochloride, polyhexanide, benzalkonium chloride, laccase and quaternary ammonium compounds effective in promoting antimicrobial property [35]	Absent in native PC. Yet, PC despite its source of derivative incorporated with lysozyme and allicin effective antimicrobial agent against *Escherichia coli, Staphylococcus aureus, Aspergillus niger* and *Candida albicans* [57]
Hemostatic agent	BC membrane derived from *Komagataeibacter* species that has been oxidized with tetramethyllpiperidine-1-oxyl shows hemostatic effects [58]	Plant-derived sodium carboxy-methyl cellulose stimulates fibrin polymerization causing aggregation of fibrins at the wound site [59]. Oxidized regenerated cellulose serves as a platform for platelet aggregation [60]. Regenerated cotton cellulose has the capability to control bleeding at the injury site [61]
Biodegradability	Slow. Animal cells are unable to cleave into β-1→4. Yet, degradation is only possible with non-enzymatic hydrolysis [39] and acid solution [62]	Slow. Due to the complicated ribbon structure arrangement and presence of impurities on nanocellulose [61,63]. A longer duration is needed to hydrolyze native PC with alkali solution [62]
Stability	High. Due to low degradation [39]	High. Due to the dense hydrogen bond in the ribbon structure arrangements of native cellulose [64]
Biocompatibility	Native BC supports human cell growth. >70% proliferation of L929 fibroblast and osteoblast cell upon being seeded on BC film [32]	Infiltration of blood vessels and infiltration of fibroblast cell was seen in native PC scaffold upon implantation, indicating bio-compatibility in the human cell [65]
Cell adhesion	Improvement of the affinity of cells towards BC is possible with the addition of nitrogen plasma. With this >95% aggregation of cells is seen [66]	Presence of hydroxyl group and specialized binding components allows site for cell adhesion in PC despite of its source of derivatives or modifications [67]
Tissue regeneration	BC incorporated with resveratrol promotes re-epithelization [68] while native BC can be utilized for tissue regeneration [49,69,70], bone tissue regeneration [50,71] and cartilage tissue [72]	Native cellulose, nanocellulose, and sodium carboxy-methyl cellulose support regeneration of tissue [53,65,73,74] and bone [75]

**Table 2:** In vivo, in vitro and clinical trial studies of plant cellulose.

Author	Material	PC Derivative	Evaluated Parameters	Type of Wound	Type of Subject	Study Design	Findings	Conclusion
Modulevsky et al. 2014 [74]	Cellulose derived from McIntosh Red apples for 3D mammalian cell culture	Hypanthium tissue of McIntosh Red apples	- Mechanical strength - Morphological analysis - Microporous study - Cell proliferation and viability	Not applicable	- Mouse C2C12 muscle myoblasts - NIH3T3 fibroblasts - Human HeLa epithelial Cells	In vitro	- Young’s modulus (*p* < 0.001) of the scaffold - High porosity in the scaffold - Clear actin stress fibers were seen - Positive adherence of the cells to the scaffold	- Increased cell proliferation of C2C12, HeLa, and NIH3T3 cells
Modulevsky et al. 2016 [65]	Cellulose derived from McIntosh Red apples	Native hypanthium tissue of McIntosh Red apples	- Histological analysis - Biocompatibility - ECM deposition - Vascularization	Incised wound	Wild-type C57BL/10ScSnJ mice	In vivo	- Infiltration of blood vessels and healthy tissue around the scaffold - Increased level of fibroblast - Reduced leukocyte after one week	- Rapid rate of re epithelization - Diminish of immune response after one week - Deposition of extracellular matrix over time
Aoshima et al. 2012 [59]	Plant derived sodium carboxymethyl cellulose for hemostasis	Not specified	Coagulation cascade	Not specified	Cephalin part of rabbit brain	In vitro and in vivo	- Activation of prothrombin - Increased formation of fibrin fibers	- Bleeding stops within minutes - No observable side effects
Singla et al. 2017 [107]	*Syzygium cumini* Cellulose Nanocrystals incorporated with AgNPs	*Syzygium cumini* leaves	- Morphological analysis - Mechanical strength - Water uptake capacity - Antimicrobial activity - Cytocompatibility - Histopathological analysis	Diabetic and acute wound	Swiss albino mice	In vitro and in vivo	- Tensile strength of cellulose nanocrystals 0.047 ± 0.005 MPa - Water absorbing capacity of 268 ± 10, 206 ± 8, and 118 ± 5% - Zone of inhibition at 40 ± 14 nm	- Entrap exudate - Provides moist surface at the sound site - Improved angiogenesis, formation of granulation tissue, deposition of Col and re epithelization
Masci et al. 2018 [108]	Plant derived oxidized regenerated cellulose (ORC) for hemostasis	Not specified	- Hemostatic duration - Follow up intervention	Laparoscopic cholecystectomy	24 patients	Clinical trial	- Controlled bleeding - Mean duration of hospital stay reduced to 2.2 days - Well tolerance toward the ORC gauze	- Bleeding controlled effectively within minutes - Absence of adverse effects

**Table 3:** In vivo and in vitro studies of bacterial cellulose.

Author	Material	BC Derivative	Evaluated Parameters	Type of Wound	Type of Subject	Study Design	Findings	Conclusion
Wen et al. 2015 [114]	BC incorporated with silver sulfadiazine nanoparticle	*Gluconacetobacter xylinus*	- Antibacterial effects in wound healing	Partial thickness wound	Wistar rats	In vitro and in vivo	- Even distribution of silver sulfadiazine nanoparticles in the BC surface was noted - Bacteriostatic effect against for E. *coli, S. aureus,* and *P. aeruginosa* was seen - Reduced bacterial count as low as 103 CFU/cm^2^ - Fresh dermis thickness was about 149µm	- Absence of infection - Significant wound size reduction by 14th day (92.35%) - Most of the wound healed on the 14th day - Early re epithelization than usual
Qiu et al. 2016 [116]	BC impregnated with vaccarin drug	*Gluconacetobacter xylinus*	- Mechanical strength - Absorption capacity - Cell viability - Inflammatory response - Microbial study	Incised wound	ICR male mice	In vitro and in vivo	- The thickness of BC impregnated with vaccarin drug exhibit a tensile strength of 459.73 ± 48.21 MPa with an elongation of 19.36 ± 10.45% - Absorption band ranges between 3200 and 3600 cm^–1^ - >80.7% cell proliferation was seen at 72 h - Better fluid retention	- Rapid wound healing, deposition of Col fibers and appearance of stratified squamous epithelium
Wu et al. 2014 [117]	BC incorporated with AgNP	Not specified	- Characterization of scaffold - Release of AgNP - Antimicrobial study - Biocompatibility	- 2nd degree deep partial-thickness wound	Wistar rat	In vitro and in vivo	- Controlled release of Ag^+^ from AgNP - The maximum bacterial reduction was seen on the 4th day which is 128.13 × 103 CFU cm^−2^ - Fibroblast proliferation was seen in the rat - Rapid migration of fibroblast to the wound site - Absence of inflammatory infiltration	- At 14th day scab fell off and growth of hair at the wound site was seen - Complete healing was seen on 21st day - Extension of epidermal tissue deeper into the wound site
Kim et al. 2013 [118]	BC nanofibrillar for TM perforation	*Gluconacetobacter xylinus*	- Characterization - TM cell proliferation - Water contact angle analysis - Histological analysis	Mechanically perforated wound	Sprague Dawley rats	In vitro and in vivo	- Tensile strength of 11.85 ± 2.43 MPa - Water contact degree of 31.17 ± 4.28; indicates hydrophilic - Transparency and non-toxic	- TM cell migration and proliferation toward BC nanofibrillar - Rapid healing was seen as early as 7 days - Auditory function was restored
Fu et al. 2012 [113]	BC for skin tissue repair	*Gluconacetobacter xylinus*	- Characterization - Mechanical strength - Evaluation of cell - Wound healing assessment	Full-thickness wound	BALB/c and C57BL/6 mice	In vitro and in vivo	- Dry BC shows a tensile strength of 10.32 MPa with an elongation of 131 MPa - The thickness of the film and the mice’s skin was the same - BC film has a large surface area with excellent porosity - A continuous layer of hASCs–BC proliferation was seen	- Rapid wound size reduction and accelerated wound healing
